# Evaluating the Efficiency of gRNAs in CRISPR/Cas9 Mediated Genome Editing in Poplars

**DOI:** 10.3390/ijms20153623

**Published:** 2019-07-24

**Authors:** Tobias Bruegmann, Khira Deecke, Matthias Fladung

**Affiliations:** Thuenen Institute of Forest Genetics, Sieker Landstrasse 2, D-22927 Grosshansdorf, Germany

**Keywords:** gRNA, homozygous editing, *Populus*, secondary structure, sequencing, transformation

## Abstract

CRISPR/Cas9 has become one of the most promising techniques for genome editing in plants and works very well in poplars with an *Agrobacterium*-mediated transformation system. We selected twelve genes, including *SOC1*, *FUL*, and their paralogous genes, four *NFP-like* genes and *TOZ19* for three different research topics. The gRNAs were designed for editing, and, together with a constitutively expressed Cas9 nuclease, transferred either into the poplar hybrid *Populus* × *canescens* or into *P. tremula*. The regenerated lines showed different types of editing and revealed several homozygous editing events which are of special interest in perennial species because of limited back-cross ability. Through a time series, we could show that despite the constitutive expression of the Cas9 nuclease, no secondary editing of the target region occurred. Thus, constitutive Cas9 expression does not seem to pose any risk to additional editing events. Based on various criteria, we obtained evidence for a relationship between the structure of gRNA and the efficiency of gene editing. In particular, the GC content, purine residues in the gRNA end, and the free accessibility of the seed region seemed to be highly important for genome editing in poplars. Based on our findings on nine different poplar genes, efficient gRNAs can be designed for future efficient editing applications in poplars.

## 1. Introduction

Since the first publications regarding application of CRISPR/Cas9 (CRISPR—**C**lustered **R**egularly **I**nterspaced **S**hort **P**alindromic **R**epeats/Cas9—**C**RISPR **As**sociated **9**) for genome editing in a plant species [1], this technique has become one of the most promising methods in molecular engineering. The leading journal “Science” called CRISPR/Cas9 the “Breakthrough of the Year 2015” [2], putting CRISPR/Cas9 on the same level with other breakthrough inventions such as the production of powdery mildew resistant wheat [3] or drought tolerant maize [4].

The CRISPR/Cas9 method is based on the *Streptococcus pyogenes* endonuclease Cas9 that causes a DNA double strand break at a specific region (reviewed in Bortesi et al. [5]). This target region is specifically recognized by a complementary guide RNA (gRNA) which forms a complex with the endonuclease. Following binding of gRNA to the target, Cas9 cuts directly upstream of a 2–6 base pair long so-called PAM (protospacer adjacent motif) sequence depending from the origin of the Cas9 nuclease. The most commonly used Cas9 from *Saccharomyces pyogenes* recognizes a PAM sequence of NGG [6]. Cas-nucleases are widely distributed in bacteria and initially involved in the defence reaction against viruses. Since the late 1980s, various CRISPR/Cas-systems have been explored in different bacterial and archaea species [7].

Following transient or stable transgenic gRNA transfer, the CRISPR/Cas9 method has been adapted to many plant [8,9], animal [10,11,12], and fungal species [13]. The idea behind the discovery was that when CRISPR/Cas9 is active in eukaryotic cells, non-homologous end-joining (NHEJ), or homologous recombination (HR) is induced as repair mechanisms of the cell following Cas9 cut. NHEJ is, however, not completely reliable and may induce insertions or deletions. Depending on the experimental design, even large donor sequences can be inserted by either NHEJ or a homology-dependent repair mechanism [5]. Relevant for the application of CRISPR/Cas9 approaches is that even one-nucleotide mutations cause a frameshift that may lead to a knockout of the targeted gene. 

The genome editing performance is strongly dependent on the gRNA sequence and its secondary structure [5]. Although general advisories for gRNA design are not consistent and often derived from human and animal cells [14], some general design parameters have been published: The GC content of the gRNA should exceed 50% [5], at least range between 30% and 80% as detected in rice [15]. Wang et al. [16] worked with CRISPR/Cas9 in human cells and recommended gRNAs targeting the non-transcribed strand for a better efficiency as well as purine residues in the last four nucleotides of the gRNA to achieve a solid binding between gRNA and Cas9. 

Secondary structure can influence gRNA effectiveness; some structural elements have been shown to have a beneficial influence on gRNA effectivity [17,18]. To analyze the secondary structure, gRNAs were designed using the bioinformatics tool RNAfold Webserver [19] by applying the Andronescu model [20] and the algorithm of Zuker and Stiegler [21] for minimum free energy (MFE) representation. Several features were mentioned to enhance gRNA effectiveness [17,18]: the last three base pairs of the gRNA (seed region) should be unpaired and freely accessible [18]; nucleotides 51–53 of the tracrRNA should be not paired to the seed region and be freely accessible, and there should be favorable nucleotides at several positions [17]. The authors found that guanidine is preferred directly after the PAM-motif (position 20), whereas cytosine was unfavorable. Besides, Doench et al. [17] found that cytosine was rejected at position 3 of the gRNA and that cytosine preferred at position 16 and guanine was rejected. Liang et al. [15] highlighted the importance of the preferably four stem loop structures in the gRNA: The RAR stem loop (GAAA) triggers the processing of the gRNA before binding to the nuclease and loops 2 (GAAA) and 3 (AGU) are required for stable complex formation. Loop 1 (CUAG) should not be related to gRNA efficiency.

**Figure 1:**
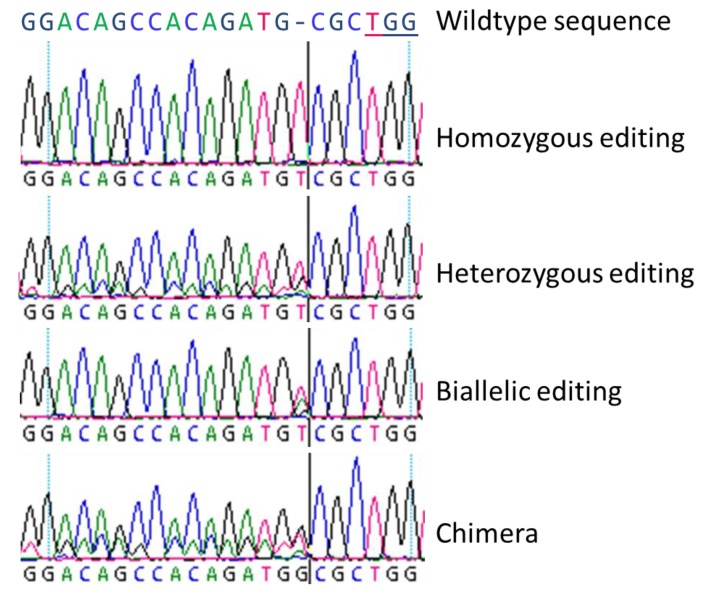
Electropherograms of four basic types of genome editing as found in poplar plants (according to Fan et al. [22]). Four basic types were obtained by Sanger-sequencing for both insertions and deletions, here as example, a single nucleotide insertion at the Cas9 cutting site: (**a**) Homozygous editing with clearly determinable curves, (**b**) heterozygous editing with overlaying wildtype sequence curves behind the cutting site of the Cas9, (**c**) biallelic editing with one overlaying peak at the edited site, and (**d**) a chimeric line with three peaks at the cutting site of Cas9 which contained two different inserted nucleotides and the wildtype and following overlaying curves. The PAM (protospacer adjacent motif) sequence TGG is underlined in the wildtype sequence.

In principle, four different genome editing types were unveiled (Figure 1) which were previously denoted by Fan et al. [22]: (a) homozygous modifications mean both alleles are edited identically, (b) heterozygous modifications are characterized by one edited allele and one wildtype-like allele, (c) in biallelic modifications both alleles are edited differently, and (d) chimeras are fusions of (partly) edited and not edited cells. Sanger-sequencing could not clarify such a special case: Two overlapping electropherograms containing one allele with the wildtype nucleotides can mean either a heterozygous mutation or a homozygous mutation as a chimeric line with wildtype tissue. In addition to the described single nucleotide insertions or deletions, CRISPR/Cas9 can be used to induce large deletions or insertion [23,24,25], inversions [26], or for gene targeting [27]. For trees, poplars served as a model species which has already been well studied with transgenic approaches since 1986 [28,29]. The first genome editing report was published by Zhou et al. [23] describing CRISPR/Cas9-based targeting of *4-COUMARATE:COA LIGASE* (*4CL*) in *Populus* × *canescens*. As *4CL* is involved in both biomass formation and flavonoid metabolism, *4CL* editing is suitable for a model approach due to its clear phenotypic aberrations. The authors achieved 100% mutational efficiency for two *4CL* copies and observed a SNP sensitivity of the CRISPR/Cas9 mechanism. In *P. tomentosa*, Fan et al. [22] employed CRISPR/Cas9 for editing of *PHYTOENE DESATURASE* (*PtoPDS*) by a transgenic approach. Mutated plants could easily be identified since knockout of *PtoPDS* led to albino plants. The obtained mutation rate of regenerated plants was 51.7%. Also Elorriaga et al. [30] showed that CRISPR/Cas9 works very powerfully and precisely in two poplar clones for generating loss-of-function *LEAFY* and *AGAMOUS* mutations by applying a transgenic system.

**Figure 2:**
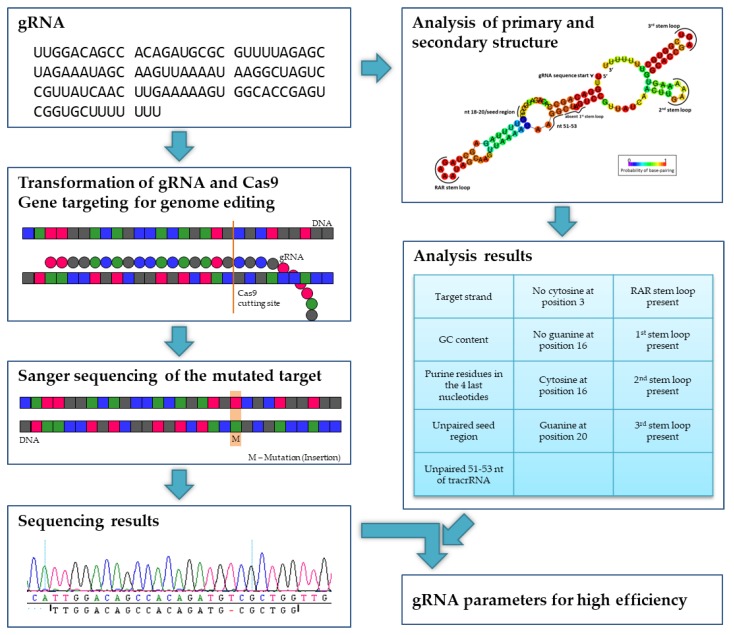
Experimental design. In poplars, a gRNA and the Cas9 nuclease were transformed. After regeneration, the target regions were sequenced and screened for mutations (in this example, an insertion). Together with the analysis of the primary and secondary structure of the gRNA, parameters for a high editing efficiency were determined.

In this paper, we report detailed analyses of the mechanism and success of CRISPR/Cas9-mediated gene editing in poplar in dependence of gRNA secondary structure (Figure 2) and, theoretically, repeated Cas9-induced editing during vegetative growth in vitro over several weeks. Following *Agrobacterium*-mediated stable CRISPR/Cas9 transfer into poplar, we aimed to edit in total 10 different genes (including paralogs), namely (i) flowering time genes *SUPPRESSOR OF OVEREXPRESSION OF CONSTANS1* (*SOC1*) and *FRUITFULL* (*FUL*) which play an additional role in biomass formation [31,32] and their paralogous genes in *Populus* × *canescens*, (ii) *NFP-like* genes which are probably associated to mycorrhization according to studies with *Lotus japonicus* [33], *Medicago truncatula* [34], and *Oryza sativa* [35], and (iii) *TORMOZEMBRYO DEFECTIVE 19* (*TOZ19*) putatively correlated with sex determination in poplar [36,37].

From the approaches with nine different gRNAs, we obtained numerous genome edited poplar plants which revealed the different editing types with single nucleotide insertions as most frequent event. In a time-series comparison, it was shown that the constitutive Cas9 nuclease did not cause secondary editings in an already edited target site. Evaluating the genome editing effort, we tried to derive a connection to the gRNA efficiency. Through gRNA secondary structure analyses, we obtained evidence that the GC content, purine residues in the last four nucleotides of the gRNA, and an at least partly unpaired seed region have an influence on gRNA efficiency for target cleavage.

## 2. Results

### 2.1. Transformation of CRISPR/Cas9-constructs Targeting Flowering Time Genes *SOC1*, *FUL*, and Their Paralogs

For our first CRISPR/Cas9 approach, we targeted *SOC1* (Potri.012G133000) and two non-annotated paralogs on chromosome 1 and 3 (*SOC1* Paralog 1: Potri.001G112400; *SOC1* Paralog 2: Potri.003G119700) as well as two paralogs of *FUL* named *AGL8.1* (Potri.008G098500) and *AGL8.2* (Potri.010G154100) which are present in the genome of *P.* × *canescens*. In total, four different gRNAs were utilized within one gRNA that targeted both existing non-annotated paralogs of *SOC1*. Multiple knockout approaches were designed to eliminate all existing gene copies, thus to reveal phenotypic changes that were not obtained in earlier RNAi approaches targeting one of these genes [38]. To date, phenotypic aberrations have not been observed. 

One transformation approach was performed to edit only the *AGL8* paralogs (N 466). Fifteen putative transgenic regenerants were obtained which were analyzed by PCR. In 12 lines, the transgenic status was verified. Transformation approach N 473 was intended to edit the three *SOC1* paralogs. Fifteen putative transgenic lines were regenerated which all revealed to be transgenic by PCR. For the genome editing of both gene groups of *SOC1* paralogs and *AGL8* paralogs, two transformation approaches (N 474 and N 477) were carried out. Regarding both, 28 regenerants were obtained. Twenty-six lines were revealed to be transgenic with the T-DNA to edit the *SOC1* group and 19 lines to edit the *AGL8* group.

Transformation line N 485 based on the transgenic clone N 473-9 was intended to add the T-DNA with the gRNAs to edit *AGL8.1* and *AGL8.2.* Here, from six putative double transgenic regenerates, four lines turned out to contain the T-DNA.

#### 2.1.1. Genome Editing of *SOC1* and Both *SOC1* Paralogs

From *SOC1* editing, we obtained 18 homozygous lines and 11 lines with a distinct biallelic editing out of 41 lines in total (Table 1). Only one of the analyzed lines was found to be unedited. 

Editing of both *SOC1* paralogs was less efficient. For *SOC1* Paralog 1 lines, at least one homozygously edited and three biallelically edited lines were obtained out of 39 in total. Fifteen of these lines had no edited target site. Most lines (19) revealed an uncertain sequencing result and were probably heterozygously edited. For *SOC1* Paralog 2, neither homozygously nor biallelically edited lines were revealed. Out of 39 lines, only three lines with further uncharacterized editing were obtained. Thirty-six of the *SOC1* Paralog 2 targets remained without any editing.

#### 2.1.2. Applied gRNAs

For knockdown of *SOC1*, gRNA1 was designed that was fully complementary to the genomic sequence in poplar clone P1. It had 60% GC content and targeted the non-transcribed strand.

The second gRNA sequence of the applied vector, gRNA2, was complementary in each nucleotide for *SOC1* Paralog 1 (Potri.001G112400, non-transcribed strand), but had an ambiguous nucleotide at position 13 regarding *SOC1* Paralog 2 (Potri.003G119700, transcribed strand). Due to this heterozygous position (nucleotides A or G, code R) within the gene Potri.003G119700 of the poplar clone P1, the gRNA did not fit perfectly to this target which could also affect editing efficiency. The gRNA2 had 40% GC content. Positive features in both present gRNAs are the avoidance of both a C at nucleotide position 3 and a G at position 16 (Table 2). Further parameters which are assigned to a negative functionality of the gRNAs are the presence of a C at nucleotide position 20 and a (partly) paired region at nucleotide position 51–53. The gRNAs differed in the number of purine residues in the last four nucleotides (two in gRNA1 and one in gRNA2).

The secondary structures of both gRNAs were analyzed with the RNAfold WebServer [19]. In both gRNAs, the seed region at nucleotide positions 18–20 was not paired to the tracrRNA in the predicted secondary structure. While the RAR stem loop as well as the second and third stem loops were present in both gRNA1 and gRNA2, the first stem loop was absent in these gRNAs.

#### 2.1.3. Genome Editing of *AGL8.1* and *AGL8.2*

The regenerated lines were Sanger-sequenced regarding *AGL8.1* and *AGL8.2*. From the *AGL8.1* editing approaches, in total 33 lines were obtained of which 13 lines were homozygously edited and 5 lines were biallelically edited. Three of the analyzed lines were not edited (Table 3).

The editing of *AGL8.2* was less efficient. Out of 31 analyzed lines, four lines were homozygously edited and three lines were biallelically edited. Fourteen lines remained without any editing in *AGL8.2*.

#### 2.1.4. Applied gRNAs

The gRNA3 was targeting the non-transcribed strand of *AGL8.1* and gRNA4 the transcribed strand of *AGL8.2*. Both gRNAs had 50% GC content and contained four purine residues in the end of the sequence (Table 3). Since gRNA4 was developed based on gRNA3 by changing three nucleotides, both gRNAs were similar concerning their secondary structure prediction by the RNAfold WebServer (Online Resource 1). The presence of RAR, first, and third stem loops as well as the avoidance of a G at nucleotide position 16 and the presence of a G at position 20 are associated with a positive effect on functionality. In contrast, a (partly) paired seed region, the absent second stem loop, the presence of a C at position 3, and the paired region at nucleotide positions 51–53 are features which are supposed to have negative effects on the functionality.

### 2.2. Transformation of CRISPR/Cas9-constructs Targeting *NFP-like* Genes

Knockout experiments were conducted to determine the function of *NFP-like* genes during mycorrhization. Therefore, two constructs were used to knockout either Paralog Pair I (*NFP-like1*, *NFP-like2*) or Paralog Pair II (*NFP-like3*, *NFP-like4*). To generate a knockout of all paralogous genes, both constructs were transformed. For the approach to modify *NFP-like1* and *NFP-like2*, 133 independent putative transgenic lines (N 481) were regenerated and tested by PCR. Of the 133 regenerants, 116 completely integrated the T-DNA in the genome. The transformation to modify *NFP-like3* and *NFP-like4* by CRISPR/Cas9 generated 56 putative transgenic regenerates (N 482). PCR amplification of the T-DNA left and right border region showed that 21 regenerants out of 56 had completely integrated the T-DNA into the poplar genome. 

#### 2.2.1. Genome Editing of *NFP-like1*

From the 116 independent transgenic lines, both CRISPR/Cas9-mediated gRNA target regions were Sanger-sequenced. Sequencing analyses showed that in all 116 PCR amplified lines only the *P. alba* allele was represented. Since *P.* × *canescens* is a hybrid of *P. tremula* and *P. alba* both alleles have to be modified to generate a complete knockout of the gene of interest. Regarding gRNA5, 42 independent regenerants out of the 116 showed a modification in the *P. alba* allele. Furthermore, 61 out of the 116 showed overlapping chromatograms indicating the more than one *P. alba* allele is in the amplified cell, which are classified as chimera. Finally, 9 out of the 116 regenerants showed no modification. No modification was observed for the gRNA6.

Selected lines have been tested a second time by amplifying the *P. tremula* allele too. Out of the seven lines tested, five also revealed modifications in the *P. tremula* allele, so that they were biallelically mutated. One out of the seven lines had the same modification as on the *P. alba* allele, thus it was homozygous. Furthermore, one line out of seven has two modified *P. tremula* alleles and so is chimeric for the gRNA5. No modifications were observed for the gRNA6 (Table 4).

#### 2.2.2. Applied gRNAs

The secondary structure of both gRNAs used to modify *NFP-like1* was calculated with the bioinformatics tool RNAfold Webserver [19] (Online Resource 1). The structural analysis of *NFP-like1* gRNA5 (targeting transcribed strand) revealed that the gRNA had a GC content of 36% and three purine residues at the end of the gRNA sequence. Furthermore, gRNA5 had a guanine nucleotide located at position 3. At position 16, the gRNA5 had an adenine nucleotide and the seed region was unpaired to the tracrRNA. Furthermore, nucleotides 51–53 belonging to the tracrRNA were accessible. While the second stem loop was absent, the remaining three stem loops were present (Table 2). The structure of *NFP-like1* gRNA6 (targeting the non-transcribed strand) is different. A cytosine is located at positions 3 and 16 of the gRNA6. Furthermore, the seed region is partly linked to nucleotides 51–53 of the tracrRNA. The gRNA6 had a GC content of 43% and no purine residues at the end of gRNA sequence. All four stem loops were present in gRNA6.

#### 2.2.3. Genome Editing of *NFP-like3* and *NFP-like4*

From the transformation of the editing vector targeting *NFP-like3* and *NFP-like4*, 21 regenerants were analyzed in respect of gRNA-target site mutations. From the 21 selected regenerants, 14 were tested with a specific primer pair for the *P. alba* allele. Unfortunately, specific amplification of the *P. tremula* allele could not be performed because the lines had been discarded before. But the remaining nine regenerants were tested with universal primers for both alleles. In Table 4, the modifications for the specific *P. alba* allele are displayed. Out of these 14 lines, seven lines revealed a heterozygous chromatogram for gRNA7. These lines were classified as chimeric since only the *P. alba* allele was amplified. The remaining seven regenerants were not modified. For gRNA8, no modification was observed in the 14 *P. alba* allele-specific analyzed regenerants. For the analysis of the remaining seven lines at both alleles, but for the target of gRNA7 two lines were classified as ambiguous and one was biallelically modified. Three regenerants out of seven were not modified, whereas one line had a homozygous modification. For this one homozygously modified line, only the *P. alba* allele was amplified even though specific primers for both alleles were used. For gRNA8 with universal primers one line out of seven showed overlapping chromatograms and was classified as ambiguous. Another line was biallelic. The remaining five lines showed no modifications.

For the gRNA7 target site in *NFP-like4*, one line revealed a homozygous modification, i.e., insertion of a single A-nucleotide, six showed overlapping sequencing electropherograms. Five out of those six lines were classified as ambiguous, while one line was biallelically modified. The remaining 13 lines were like the wildtype. Except of one regenerant with a not nearer determined editing event, all analyzed plants were genetically like wildtype regarding the gRNA8 targeted DNA region.

#### 2.2.4. Applied gRNAs

The structural analysis of gRNA7 (targeting the non-transcribed strand of *NFP-like3* and the transcribed strand of *NFP-like4*) revealed that an adenine nucleotide is located at position 3 (Table 2). At position 16, the gRNA7 had a guanine nucleotide and the seed region was paired to the tracrRNA. Furthermore, nucleotides 51–53 belonging to the tracrRNA were linked to the tracrRNA. The gRNA7 had a GC content of 52% and two purine residues at the end of the gRNA sequence. The gRNA formed presumably the RAR, first, and second stem loop. The third stem loop was absent. The gRNA8 (targeting *NFP-like3* and *NFP-like4* not transcribed strand) had a GC content of 30% and two purine residues at the end of gRNA sequence. Computed structure of gRNA8 showed a cytosine nucleotide located at positions 3 and 16. Furthermore the seed region was partly linked to nucleotides 51–53 of the tracrRNA. The RAR, second, and third stem loop of gRNA8 were present, but the first stem loop was absent.

#### 2.2.5. CRISPR/Cas9 Cleavage Over Time 

In randomly performed gRNA target site sequencing of vegetatively propagated plants from the *NFP-like* gene editing approaches, we found no further activities of the Cas9 after gene editings occurred at the specific gRNA target sequence. To analyze potential secondary modifications by CRISPR/Cas9, DNA was extracted seven months after first sequencing analysis. DNA was extracted and gRNA Target Sites 1 and/or 2 sequencing of selected plants were performed. For *NFP-like1*, we found in five out of six lines the same modifications like the first time (Online Resource 2). Only one line, already noted as a chimera, showed a biallelic status. In case one allele that was twice modified in this line showed lower peaks in the chromatogram, it could be that this was just underrepresented in the tissue sample from which the DNA was extracted. Secondly, we were not able to observe any further modifications between the first and second sequencing regarding the specific gRNA target sequence. 

In addition, we tested four of the N 482 regenerated lines. No further modifications were observed for both gRNA targeting sites, neither for *NFP-like3* nor *NFP-like4*. For *NFP-like3* we only amplified the *P. alba* allele with the used primers, so that we only could observe modifications for one allele.

### 2.3. Modification of *TORMOZEMBRYO DEFECTIVE 19* (*TOZ19*)

For the knock-out approach of *TOZ19*, we obtained 23 independent putative transgenic lines (N 470). PCR amplification of the hygromycin resistance gene and the Cas9 nuclease each displayed 15 positive lines. From all 15 independent transgenic lines, the gRNA-mediated CRISPR/Cas9 target region 9 was PCR amplified and 13 lines revealed PCR-amplified bands of expected size which were Sanger-sequenced. Out of these, five lines showed homozygous mutations (two insertion of C, one insertion of T, one deletion of T, one deletion of 9 bp), three lines revealed overlapping electropherograms, four lines were similar as wildtype, and one line failed to amplify (Table 5).

#### Applied gRNA

Structural analysis of *TOZ19*-targeting gRNA9 (targeting non-transcribed strand) showed that at position 3 a cytosine nucleotide was located that should be avoided (Table 2). Furthermore, adenine nucleotides were at both positions 16 and 20, in both positions other nucleotides are favored. In addition, the seed region is partly paired to the nucleotides 51–53, thus both regions are not freely accessible. The gRNA9 had a GC content of 55% and two purine residues at the end of gRNA sequence. All four stem loops are present in gRNA9, but the first stem loop only partly.

### 2.4. Overall Homozygous Editing

Summarizing all different approaches, we obtained 44 homozygously edited lines (Table 6). Out of them, 31 lines revealed a single nucleotide insertion of A or T. Nine lines showed an insertion of C or T, and only four lines showed deletions of either a single nucleotide or larger sequences.

## 3. Discussion

Genetics and genomics of *Populus* received attention in many laboratories throughout the world in the past 30–40 years since this genus has become a model system for forest tree species [28,29,39,40]. Trees play a key role in many terrestrial eco-systems as they are involved in valuable biogeochemical cycles (water, oxygen, and nitrogen). Forests are highly important for carbon sequestration and regulation of CO_2_ concentration. In the beginning of the genomics area, basic science was feasible with tree species such as poplar and eucalyptus with small genomes, but challenging with species like beech, oak, spruce, or pine. These species have huge genome sizes with DNA full of retrotransposons, and characterized by large intron sizes and a high number of mini- and microsatellite repeats [41]. The poplar genome is with about 550 Mbp relatively small in size [42], and complete genome sequences are available for *P. trichocarpa* [42], *P. euphratica* [43], *P. tremula* [44], *P. deltoides* (https://phytozome.jgi.doe.gov/pz/portal.html #!info?alias=Org_PdeltoidesWV94_er), and partially for *P. pruinosa* [45] and *P. alba* [43].

All different poplar genomes are very similar and collinear to each other, but with particular features like higher number of salt tolerance genes in *P. euphratica* [43]. For *P. trichocarpa*, [42] assumed a quite recent whole-genome duplication event (about 60–65 Mya ago), resulting in about 8000 pairs of duplicated genes. Thus, for many genes, including *SOC1, FUL*, *NFP-likes*, and *TOZ19*, a paralogous copy is available in the *P. trichocarpa* genome. This also holds true for the poplar clones used for transformation in this study, INRA 717-1B4 (*P.* × *canescens*; [23,46,47,48]) and W52 (*P. tremula*; [44,49]). Thus, to obtain clear knockout lines for both functional and physiological analyses, all paralog copies should be targeted by the CRISPR/Cas9 approach.

In poplar, few studies on gene editing by CRISPR/Cas9 have been published in the last four to five years [22,23,30,50,51,52,53]. All studies have in common that CRISPR/Cas9 has successfully been applied to insert a mutation in one or more genes for knockout (KO) of the poplar target gene. [53] knocked out all *NST/SND* orthologs and confirmed their central role in the secondary cell wall (SCW) formation in wood fibres, xylem ray parenchyma cells and phloem fibers in hybrid aspen. [50] discovered an epigenetic mechanism that modified anthocyanin biosynthesis in poplar. Following knockout of a histone H3K9 demethylase gene *JMJ25*, *MYB182* expression is altered leading to a change of the histone methylation status in the chromatin. Muhr et al. [51] knocked-out the two transcription factors involved in shaping plant architecture, *BRANCHED1,* and *BRANCHED2*, and found that in contrast to *Arabidopsis*, in poplar *BRANCHED2* plays an even more critical role in bud outgrowth regulation.

By reviewing the current state of CRISPR technology for genome editing in forest, fruit, and nut trees, Bewg et al. [54] emphasize the importance of the high efficacy of setting homozygous knockout mutations to avoid laborious multigenerational crosses. 

### 3.1. Biallelic and Homozygous Editings

In general, we observed all three basic types of modifications as described by Fan et al. [22] by Sanger-sequencing, and we could clearly distinguish homozygous, biallelic, and chimeric lines. Heterozygous mutations could not be revealed by simple Sanger-sequencing since the obtained sequencing results are very similar to chimeric lines with a mix of wildtype and homozygously mutated sequences.

For the nine DNA targets in the twelve different genes included in this study, in total we found 30 biallelic and 44 homozygous editing-based mutations, varying between the different genes. Both, the highest number of biallelic and homozygous editing were found in gene *SOC1.* However, for three of the twelve DNA targets neither biallelically nor homozygously edited lines were revealed. Especially homozygous genome editing is of special interest in trees, where either long vegetative phases exist or, as in poplar, selfing of parent lines is impossible due to dioecy. As shown for the eight DNA targets under study here, homozygous genome editing occurred and in the majority of cases, by insertion or deletion of just one nucleotide, resulting in a frame shift of the coding region. This causes a more or less precocious stop-codon in the predicted transcript sequence, leading to an incomplete protein.

The sequences revealed the preferable insertion of A/T, that was also reported for plants by Bortesi et al. [5]. Nucleotide substitutions have not been revealed in all approaches which are published here. This is in accordance with earlier studies [15] which were summarized by Bortesi et al. [5], which revealed only rare substitution events except in the case of soybean protoplasts [55].

### 3.2. gRNA Efficiency

Since Doench et al. [17] found that gRNA structure elements have an impact on cleavage efficiency, a structural analysis was performed with each gRNA to verify the assumption that the tested gRNAs differ in their activity. A GC content between 40% and 60% and purine residues in the four last nucleotides of the gRNA seem to support the editing efficiency argument of [3]. Also in the perennial *Vitis vinifera*, a high GC content improved the CRISPR/Cas9-mediated editing efficiency [56]. For gRNA secondary structure calculation the bioinformatical tool RNAfold Webserver [19] was used that is based on the minimum free energy (MFE) algorithm from Zuker and Stiegler [21] and that utilized the RNA folding parameters from the Andronescu model, 2007 [20]. Doench et al. [17] unveiled several determined gRNA locations with an influence on the editing efficiency: Guanine was preferred at nucleotide position 20 of the gRNA and a cytosine was adverse. Furthermore, they reported that no cytosine should be located at position 3, but a cytosine is preferred at position 16, while a guanine at this location has a disadvantageous impact on gRNA efficiency. Wong et al. [18] further analyzed the dataset of Doench et al. [17] and reported that nucleotides at position 18–20 are the seed region of the gRNA. This region and the nucleotides located at 51–53 should be unbound and accessible to form efficient gRNAs. If the seed region would bind to position 51–53 of the gRNA, it would be non-functional. The findings from both of these publications can be correlated with the efficiency of the used gRNAs. Comparing between the two DNA single strands, the non-transcribed DNA strand should be targeted by the gRNA for an enhanced efficiency [3]. 

For genome editing of the well-known flowering time-determining genes *SOC1* and *FUL,* including their paralogous genes, four different gRNAs were designed and integrated by a transgenic approach into poplar plant cells. The obtained transgenic lines were Sanger-sequenced and analyzed in relation to the gRNA efficiency factors mentioned above. For these four gRNAs, it was notable that both gRNA1 and gRNA3 which were targeting the non-transcribed strand of the intended gene worked with higher efficiency than both gRNA2 and gRNA4 which were targeting the transcribed strand. This effect was previously observed in CRISPR/Cas9 approaches in human cells [16].

The gRNA1 for the editing of *SOC1* from poplar clone P1 worked with the highest efficiency. In contrast to the other three gRNAs (gRNA2-4), gRNA1 had the highest GC content (60%) and only partly paired nucleotides 51–53, and gRNA2 revealed the lowest efficiency in this approach. This is comprehensible regarding the target *SOC1* Paralog 2, since the gRNA sequence was not completely complementary to the genomic sequence, but contained a heterozygous SNP at position 13 of the gRNA. Regarding target *SOC1* Paralog 1, the editing efficiency is indeed better in comparison to Paralog 2 since the gRNA sequence is 100% complementary to the genomic sequence. Compared to gRNA1, the lower efficiency of gRNA2 could be found in the paired nucleotide region 51–53, only one purine residue in the last four nucleotides, and a lower GC content. However, the GC content is still within an acceptable range (40%).

Both gRNA3 and gRNA4 work with a lower efficiency than gRNA1. Between the *AGL8* paralogs targeting gRNAs, gRNA3 had a higher efficiency than gRNA4 although the seed region was paired in gRNA3. In gRNA4, the seed region was only partly paired and all other parameters were identical to gRNA3 (except the target strand). Thus a decreasing editing efficiency by a paired seed region mentioned by Wong et al. [18] could not be verified here. 

*NFP-like* genes were selected to investigate their impact in poplar to enhance mycorrhization. In *P. trichocarpa*, four genes seemed to have an impact on mycorrhizal formation, named as *NFP-like1*, *NFP-like2*, *NFP-like3*, and *NFP-like4*. Since the poplar genome was recently duplicated [42], we had to target two genes simultaneously to obtain a CRISPR/Cas9 induced knockout of the genes. Therefore, we searched for target sequences that were similar in both paralog genes (Pair 1: *NFP-like1* and *NFP-like2*, Pair 2: *NFP-like3* and *NFP-like4*). For each paralog pair, we used two target sites to enhance CRISPR/Cas9 modifications. Three knockouts were used to determine which of the genes best supports mycorrhiza formation; two single knockouts of the paralog genes and a knockout of all *NFP-like* genes. We found that the tested gRNAs differ in their modification rate. For the Paralog Pair 1 containing *NFP-like1* with two gRNAs, we only observed modifications in Target 1. Structural analyses were performed for all gRNAs used under study. Beneficial structures are displayed in Table 2.

The gRNA with the highest editing efficiency was gRNA5 in *NFP-like1*, the beneficial structures of gRNA5 are that it had a freely accessible seed region and the tracrRNA is unbound at position 51–53. Furthermore, at position 3 of gRNA5 is no cytosine and at position 16 no guanine, which are unfavorable. A guanine is located at position 20 of gRNA5, and three out of four nucleotides at the end of the gRNA sequence are purine residues. On the other hand, the gRNA that did not reveal any mutation in the analyzed regenerates was the second *NFP-like1*-gRNA6. In this gRNA, one possibility that gRNA6 did not reveal in any modification, could be that the nucleotide 19 of the seed region is paired to nucleotide 51 of the tracrRNA. According Wong et al. [18], gRNAs that have a paired seed region with nucleotides 51–53 of the tracrRNA were correlated with non-functional gRNAs. In case that gRNA9 had a paired seed region and generated modifications we cannot confirm this finding for the gRNAs used under study. In addition, gRNA6 had no purine residues at the last four nucleotides, so that this can be correlated with non-effective gRNA [16] and the target strand is the transcribed strand. Although gRNA7 had a bound seed region to the tracrRNA too, modifications were observed at the target. This may be correlated with the target strand, that is the not transcribed strand and with the presence of two purine residues at the end of gRNA7. The used gRNA8 showed sporadic modifications even though it had a paired seed region and paired tracrRNA nucleotides 51–53. In addition, gRNA8 had a lower GC content of 30%, but it still generates modifications. According to gRNA6, it shows that gRNA8 had two purine residues at the end of gRNA sequence. These finding may indicate that we had problems generating a knockout mutant for *NFP-like3* and *NFP-like4*. 

For the editing of the aspen-sex marker gene *TOZ19*, gRNA9 was transformed into poplar clone W52. From a total of 13 lines generated, five lines were homozygously edited, but a further five lines didn’t reveal any editing in the target region. Given the gRNA properties, this result is remarkable because the seed region was partially bound to the region of nucleotides 51–53. According to Wong et al. [18], this could be an exclusion criterion for functional gRNAs. However, either possibly the inadequate binding of the seed region or the given positive properties of the gRNA (55% GC content plus two purine residues in the last four nucleotides) led to functional integrity.

The significance of the individual required nucleotides in the gRNA sequence proposed by Doench et al. [17] could not be confirmed. On the contrary, e.g., the required cytosine at position 16 did not seem to have any influence. The gRNAs1, 3, and 5 worked very well but contained no cytosine here. The only gRNA with cytosine at this position was gRNA6, which did not work at all. It cannot be ruled out, however, that the functionality can be influenced by this nucleotide composition on a small scale if all other properties are suitable. In practice, CRISPR/Cas9 is a widely used tool, because apart from an NGG sequence as PAM, no essential requirements are placed on the target sequence. 

The presence of gRNA stem loops that was highlighted by Liang et al. [15] in regard to a cleavage effort could not be verified. All gRNAs contained the RAR stem loop classified as crucial. Differences in the gRNAs allowed conclusions to be drawn regarding the stem loops 1–3: Since gRNA1 worked well without stem loop 1 and gRNA3 without stem loop 2, they could not be crucial for function. The 3^rd^ stem loop was only absent in gRNA7 that didn’t work satisfactorily, but contained further adverse features, thus the importance of loop 3 could not be clearly determined.

Taking our results together, particular attention should be paid to the GC content of the gRNA, as also recommended by Liang et al. [15] and Ren et al. [56], and the need for four purine residues in the last four positions of the gRNA according to Wang et al. [16], based on their findings in human cells. In agreement with the gRNA sequence, additional attention should then be paid to target the non-transcribed DNA strand and to possible bonds (for example to the seed region) when inserted into the tracrRNA that can be designed by the experimenter. 

### 3.3. No On-Going Modifications

Control of Cas9 by a constitutive promoter as in the present approaches could lead to persistent nuclease activity and, thus, to subsequent editings over time at the specific gRNA target sequence. To identify possible additional modifications after the first editing by the applied CRISPR/Cas9 system, we extracted DNA from selected lines of edited *NFP-like* genes again sometime after the first editing-analysis. The second editing-analysis was performed seven months (with one plant per line) after the first analysis, respectively. All analyzed lines were vegetatively propagated from those plants which were originally analyzed for editing. During analysis of the *NFP-like1* chromatograms we detected no further modifications in six out of the seven tested lines, while the 7^th^ was determined to be a chimera. Following analyses of four regenerates from a second transformation (N 482), similar editings were found in the second analyses as in the first analyses. 

Taking all these results together, the CRISPR/Cas9 mediated editings in the selected regenerants were not additionally modified after seven months of vegetative propagation of in vitro culture. Additional modifications in plants for the targets of *NFP-like1* gRNA5, *NFP-like3* gRNA7 and gRNA8 as well as *NFP-like4* gRNA7 and gRNA8 have not been detected. Over all lines that were tested we did not find any further modifications at the specific gRNA target sites used for analysis, even though the Cas9 have been expressed under a constitutive promotor.

### 3.4. Perspecives

The results presented in this study confirm the high editing efficiency of the CRISPR/Cas9-approach as well as the stability of the original mutation over several months in a transgenic background, even when the Cas9 was under the control of a constitutively expressed promoter. However, given the persistent societal skepticism towards transgenic plants, further potential of CRISPR/Cas9 applications is provided in DNA-free approaches. Transferring just ribonucleic–protein complexes into plant cells for transient genome editing would lead to genome edited plants without transgenic DNA integration. These plants would be free of T-DNA border sequences, resistance marker genes, or the Cas9 gene. One important future challenge is to transfer the CRISPR/Cas9 system developed for poplar to other forest tree species without well-developed in vitro regeneration systems. Possible approaches could be in planta or cuttings CRISPR/Cas9-applications, or ovule or pollen modification. This is even more important as the ensured survival of forest trees is crucial because they are highly endangered by abiotic and biotic stresses as a consequence of the ongoing climate change. Due to the low domestication rate of forest trees, the available high genetic diversity can be used for future-oriented breeding goals. However, conventional breeding takes many years or decades due to the long generation cycles of tress, while the climate change proceeds faster, fortunately genome editing is able to accelerate the breeding process, for instance to induce pest resistance or tolerances against abiotic stresses.

## 4. Materials and Methods 

### 4.1. In Vitro Cultivation

The well-known *P.* × *canescens* poplar clone INRA 717-1B4 (P1) [46] was used for *SOC1*, *FUL*, and their paralogous genes as well as the *NFP-like* genes. The *P*. *tremula* clone W52 already transformed with the early-flowering construct HSP::At*FT* [57] was used for *TOZ19* knock-out transformation experiments. P1 and W52 were cultivated and propagated in vitro on McCown Woody Plant Medium (WPM) (Duchefa Biochemie, Haarlem, The Netherlands), in Magenta containers (Sigma-Aldrich, St. Louis, MO, USA). Plants had 23 °C, RH 50% (± 5%), and 24 h light with 18 µmol photons m^–2^ s^–1^.

### 4.2. Vector Design

For all genes to be targeted by the different approaches, sequences were first taken from *P. trichocarpa* genome sequence v3.0 (https://phytozome.jgi.doe.gov/pz/portal.html#!info?alias=Org_Ptrichocarpa) and adapted to the respective *P*. × *canescens* sequences (https://urgi.versailles.inra.fr/Species/Forest-trees/Populus/Clone-INRA-717-1B4). Cas9, gRNAs, and the selection marker genes were each driven by 35S and U6 promoters.

The transformation vectors (Table 7, Online Resource 3) contained a T-DNA region with one or two open reading frames for the gRNA sequences (Online Resource 1), Cas9 sequence (Online Resource 3), as well as the selection markers *NptII* for kanamycin resistance or *HptII* for hygromycin resistance.

For the *SOC1* and *FUL* knockdown approach, two transformation vectors were designed with each two different gRNAs. Vector B798p6ioR-Cas-SOC encodes the gRNA for *SOC1* (gRNA1) and the gRNA for both *SOC1* paralogs that should be knocked out with the same gRNA (gRNA2) due to their sequence similarity. The second vector B797p9ioR-Cas-AGL-2 encodes for two gRNAs, each targeting one *FUL* paralog (gRNA3 for *AGL8.1* and gRNA4 for *AGL8.2*). 

For the genetic analysis of *NFP-like* genes which are thought to have an impact on mycorrhiza formation two vectors were designed containing two gRNAs each. C234p6ioR-35sCasWToi-P57 encodes two gRNAs (gRNA5, gRNA6) for *NFP-like1* and *NFP-like2*. Furthermore, the vector C235p9ioR-35sCasWToi-P810 encodes two gRNAs (gRNA7, gRNA8) to target *NFP-like3* and *NFP-like4* simultaneously.

The B796p6ioR-35sCWT plasmid contains gRNA9 targeting the *TOZ19* gene in Exon 3. The plant resistance marker hygromycin is driven by the 35S promoter and interrupted by the intron of the *RBCS small subunit* gene from potato. 

### 4.3. Genetic Transformation

*Agrobacterium*-mediated genetic transformations of poplar plants were performed by applying a leaf-disc transformation protocol [58] or with an advanced leaf disc method as described in Bruegmann et al. [59]. In summary, for the latter, plant tissue was infected with *Agrobacterium* and washed with Cefotaxime (500 mg/L, Duchefa Biochemie) and cultivated on WPM medium as described in Fladung et al. [58] and Bruegmann et al. [59], with thidiazuron added to a final concentration of 0.0022 mg/L ([60], Duchefa Biochemie). For selection, the regeneration medium was supplemented with Cefotaxime (500 mg/L, Duchefa Biochemie) and kanamycin (50 mg/L, Duchefa Biochemie) or hygromycin (20 mg/L, Duchefa Biochemie), depending on the selection marker gene. Until beginning stem regeneration, the batches were cultivated in the dark. Once regenerating shoots began to appear, the regenerants were transferred for one month to low light condition with 2.5 µmol photons m^−2^ s^−1^ until initial multiple stem formation and later on under standard conditions as described above. Selected regenerants were used for molecular analyses. After verification of transgenic status, the plants were propagated on WPM medium without supplements [38].

### 4.4. DNA Extraction and PCR

Genomic DNA from poplar leaves was isolated according the ATMAB dependent protocol of Dumolin et al. [61]. Plant material was grinded with a swing mill (Retsch MM300, Retsch, Haan, Germany) as described by Bruegmann and Fladung [32].

To verify the transgenic status of the putative transgenic regenerants, PCR amplifications were carried out under standard conditions and separated by TBE-agarose gel electrophoresis [62]. Here, PCR primer pairs (each 5′–3′) were used which were suitable for all transformation vectors for both selection markers *NptII* (forward: TTG GGT GGA GAG GCT ATT CGG; reverse: GAA GGC GAT AGA AGG CGA TGC, or forward: TTG AAC AAG ATG GAT TGC ACG; reverse: AAG AAG GCG ATA GAA GGC GA) or *HptII* (forward: GAG AAG TTT GAT AGC GTG TCT G; reverse: TAG CGT CAC AGC GGC CTT G), respectively, and partly for *Cas9* endonuclease (forward: GCT CCA GAC AAG AAG TAC AGC; reverse: TGT TCA CGC GAA GGA TGT CG) and part of the gRNA sequence (forward: TCA AAA GGC CCC TGG GAA TC; reverse: AAA AAA GCA CCG ACT CGG TG). For sequencing of edited target regions, the PCR primers given in Online Resource 4 were used.

### 4.5. Sample Collection for Time Series

In the approach used here to knockout *NFP-like* genes, a time series to unravel putative CRISPR/Cas9 repeated gene editing activity was established. For CRISPR/Cas9 analyses, plant material was collected for DNA extraction at different points in time. For the first analysis six months after transformation, shoot tips from regenerating explants were cut and the lower part of the explant was used for DNA extraction while the upper part was used for rooting and subsequent vegetative growth. Propagation was performed using the upper part of the analyzed plant material. To investigate possible differences between propagated plants, 11 months (seven months after the first sequencing) after transformation already analyzed plants were again sampled. For each independent regenerated transformation event, we chose one explant to verify the identical CRISPR/Cas9-set mutation in all propagated plants. 

### 4.6. Sequence Analyses 

Amplified PCR products were mixed with one of the forward and reverse sequencing primers (Online Resource 4). Sanger sequencing reactions were performed by StarSEQ (Mainz, Germany). All sequence analyses were performed with SeqMan Pro 15 (DNASTAR, Madison, WI, USA).

### 4.7. gRNA Analysis

The secondary structures of the applied gRNAs (Online Resource 1) were analyzed with the RNAfold WebServer [19], accessible under http://rna.tbi.univie.ac.at/cgi-bin/RNAWebSuite/RNAfold.cgi (05/06/19). This software relies on the RNA folding model of [20] and the minimum free energy (MFE) computing algorithm of [21].

## Figures and Tables

**Table 1:** Numbers of modifications in *SUPPRESSOR OF OVEREXPRESSION OF CONSTANS1 (SOC1)*, *SOC1* Paralog 1, and *SOC1* Paralog 2. Abbreviations: hom—homozygous editing, bial—biallelic editing, Chim—Chimera, ambi—edited but ambiguous, w/o—without editing/unmodified, ∑—sum of tested lines.

	gRNA1 (*SOC1*)						gRNA2 (*SOC1* Paralog 1)						gRNA2 (*SOC1* Paralog 2)					
Plant line	hom	bial	Chim	ambi	w/o	∑	hom	bial	Chim	ambi	w/o	∑	hom	bial	Chim	ambi	w/o	∑
**N 473**	4	6		5		15	1			7	5	13				2	12	14
**N 474**	6	2		2	1	11		2	1	5	3	11					11	11
**N 477**	8	3	1	3		15		1		7	7	15				1	13	14
**Sum**	18	11	1	10	1	41	1	3	1	19	15	39	0	0	0	3	36	39

**Table 2:** Selected features with a possible influence on the efficiency of the applied gRNAs.

Feature	gRNA1	gRNA2	gRNA3	gRNA4	gRNA5	gRNA6	gRNA7	gRNA8	gRNA9
**Target gene**	*SOC1*	*SOC1* Paralogs 1/2	*AGL8.1*	*AGL8.2*	*NFP-like1*	*NFP-like1*	*NFP-like3*/*NFP-like4*	*NFP-like3*/*NFP-like4*	*TOZ19*
**Target strand**	non-transcribed strand	(non-)transcribed strand^1^	non-transcribed strand	transcribed strand	transcribed strand	non-transcribed strand	(non-)transcribed strand^2^	(non-)transcribed strand^2^	non-transcribed strand
**GC content**	60%	40%	50%	50%	36%	43%	52%	30%	55%
**Purine residues in the 4 last nucleotides**	2 (GCGC)	1 (UCAC)	4 (AGGG)	4 (AGGG)	3 (AATG)	0 (CTTT)	2 (ATTG)	2 (CATA)	2 (GCCA)
**Unpaired seed region**	yes	yes	no, paired	no, partly paired (19–20)	yes	no, partly paired (19–20)	no, paired	no, partly paired (19–20)	no, partly paired (18–19)
**Unpaired 51–53 nt of tracrRNA**	no, partly paired (52–53)	no, paired	no, paired	no, paired	yes	no, partly paired (51–52)	no, paired	no, paired	no, partly paired to seed region
**No cytosine at position 3**	yes (G)	yes (A)	no (C)	no (C)	yes (U)	no (C)	yes (A)	no (C)	no (C)
**No guanine at position 16**	yes (U)	yes (U)	yes (A)	yes (A)	yes (A)	yes (C)	no (G)	yes (U)	yes (A)
**Cytosine at position 16**	no (U)	no (U)	no (A)	no (A)	no (A)	yes (C)	no (G)	no (U)	no (A)
**Guanine at position 20**	no (C)	no (C)	yes (G)	yes (G)	yes (G)	no (U)	yes (G)	no (A)	no (A)
**RAR stem loop present**	yes	yes	yes	yes	yes	yes	yes	yes	yes
**1^st^ stem loop present**	no	no	yes	yes	yes	yes	yes	no	partly
**2^nd^ stem loop present**	yes	yes	no	no	no	yes	yes	yes	yes
**3^rd^ stem loop present**	yes	yes	yes	yes	yes	yes	no	yes	yes

^1^ gRNA2 is targeting the non-transcribed strand of *SOC1* Paralog 1 and the transcribed strand of *SOC1* Paralog 2. ^2^ Both gRNA7 and gRNA8 are targeting the non-transcribed strand of *NFP-like3* and the transcribed strand of *NFP-like4*.

**Table 3:** Numbers of modifications in both genes *AGL8.1* and *AGL8.2*. Abbreviations: hom—homozygous editing, bial—biallelic editing, Chim—Chimera, ambi—edited but ambiguous, w/o—without editing/unmodified, ∑—sum of tested lines.

	gRNA3 (*AGL8.1*)						gRNA4 (*AGL8.2*)					
Plant line	hom	bial	Chim	ambi	w/o	∑	hom	bial	Chim	ambi	w/o	∑
**N 466**	1	2		4	3	10	1			2	8	11
**N 474**	5			4		9		1		4	3	8
**N 477**	5	2	2	1		10	2	2		3	2	9
**N 485**	2	1		1		4	1		1	1	1	4
**Sum**	13	5	2	10	3	33	4	3	1	10	14	31

**Table 4:** Numbers of modifications in *NFP-like* genes. Abbreviations: hom—homozygous editing, bial—biallelic editing, Chim—Chimera, ambi—edited but ambiguous, w/o—without editing/unmodified, ∑—sum of tested lines.

		**gRNA5 (*NFP-like1*)**						**gRNA6 (*NFP-like1*)**					
**Plant line**	**Gene**	**hom**	**bial**	**ambi**	**Chim**	**w/o**	**∑**	**hom**	**bial**	**ambi**	**Chim**	**w/o**	**∑**
**N 481**	*NFP-like1*	1	5	0	1	0	7	0	0	0	0	7	7
		**gRNA7 (*NFP-like3*/*NFP-like4*)**						**gRNA8 (*NFP-like3*/*NFP-like4*)**					
**N 482**	*NFP-like3*	0	1	2	0	3	7	0	1	1	0	5	7
**N 482**	*NFP-like4*	1	1	5	0	14	21	0	0	1	0	20	21

**Table 5:** Numbers of modifications in *TOZ19*. Abbreviations: hom—homozygous editing, bial—biallelic editing, Chim—Chimera, ambi—edited but ambiguous, w/o—without editing/unmodified, ∑—sum of tested lines.

	gRNA9 (*TOZ19*)					
Plant line	hom	bial	Chim	ambi	w/o	∑
**N 470**	5	0	0	3	5	13

**Table 6:** Detailed view on the distribution of homozygous editings. Focused on the revealed 44 homozygous target editings, the most frequent event was the insertion of A or T. Single base deletions or found deletions larger than three nucleotides occurred rarely. For the editing of *SOC1* Paralog 2 and *NFP-like3*, no homozygous mutations have been obtained.

	*SOC1*	*SOC1* Paralog 1	*AGL8.1*	*AGL8.2*	*NFP-like1*	*NFP-like4*	*TOZ19*	Sum
**Insertion A**	2		12	4		1		19
**Insertion T**	11						1	12
**Insertion C**							2	2
**Insertion G**	5	2						7
**Deletion T**					1		1	2
**Deletion of > 3 nucleotides**			1				1	2

**Table 7:** Target genes, gRNA sequences, and transformation vectors. Regarding *SOC1* paralog 2, the gRNA2 was not completely complementary to the target. The target region had one heterozygous nucleotide at position 13 (C/T). All other given gRNAs were 100% complementary to their respective target. If the target genes had no official alias name in the genome database of *Populus trichocarpa*, the name is put in double quotes. The given PAM (bold, underlined) motif is not part of the gRNA in the transformation vectors.

gRNA No	Target Gene	Alias	gRNA Sequence + PAM Motif	Plasmid (Agrobacterium Number; Internal Code)
1	Potri.012G133000	*SOC1*	TTGGACAGCCACAGATGCGC**TGG**	B798p6ioR-Cas-SOC (A253)
2	Potri.001G112400	“*SOC1* paralog 1”	GTAAATGCATCTTCCTTCAC**AGG**	B798p6ioR-Cas-SOC (A253)
	Potri.003G119700	“*SOC1* paralog 2”	GTAAATGCATCTTCCTTCAC**AGG**	B798p6ioR-Cas-SOC (A253)
3	Potri.008G098500	*AGL8.1*	TTCCCGAATGAGTTCAAGGG**CGG**	B797p9ioR-Cas-AGL-2 (A251)
4	Potri.010G154100	*AGL8.2*	TGCCCGAATGTGTTTAAGGG**CGG**	B797p9ioR-Cas-AGL-2 (A251)
5	Potri.005G128400	*“NFP-like1”*	AGTTGATTTGGAAATAATG**GGG**	C234p6ioR-35sCas (A259)
6	Potri.005G128400	*“NFP-like1”*	TTCTTCTCGATTCCACATTT**CGG**	C234p6ioR-35sCas (A259)
7	Potri.008G160600	*“NFP-like3”*	CGAGAAAAGGTCACCGATTG**AGG**	C235p9ioR-35sCas (A260)
	Potri.010G078700	*“NFP-like4”*	CGAGAAAAGGTCACCGATTG**AGG**	C235p9ioR-35sCas (A260)
8	Potri.008G160600	*“NFP-like3”*	TACTTGGTTTTGATATCATA**AGG**	C235p9ioR-35sCas (A260)
	Potri.010G078700	*“NFP-like4”*	TACTTGGTTTTGATATCATA**AGG**	C235p9ioR-35sCas (A260)
9	Potri.019G047300	*TOZ19*	TCCAGAAGCATGGCAAGCCA**TGG**	B796p6ioR-35sCWT (A255)

## Supplementary Materials

Supplementary materials can be found at https://www.mdpi.com/1422-0067/20/15/3623/s1. Online Resource 1: Transformation vectors; Online Resource 2: gRNA sequences and structures; Online Resource 3: PCR and sequencing primers; Online Resource 4: Sequencing results of *NFP-like1* at first time point.

## Abbreviations

*4CL*	*4-COUMARATE:COA LIGASE*
*AGL*	*AGAMOUS-LIKE*
ATMAB	Alkyltrimethylammonium bromide
CRISPR	Clustered Regularly Interspaced Short Palindromic Repeats
*FUL*	*FRUITFULL*
gRNA	guideRNA
*HptII*	*HYGROMYCIN PHOSPHOTRANSFERASE II*
HR	homologous recombination
MFE	minimum free energy
*NFP*	*Nod Factor Perception*
NHEJ	non-homologous end-joining
*NptII*	*NEOMYCIN PHOSPHOTRANSFERASE II*
*P.*	*Populus*
PAM	protospacer adjacent motif
*PtoPDS*	*PHYTOENE DESATURASE* from *Populus tomentosa*
RH	relative humidity
RNAi	RNA interference
*SOC1*	*SUPPRESSOR OF OVEREXPRESSION OF CONSTANS1*
TBE	Tris/Borate/EDTA
*TOZ19*	*TORMOZEMBRYO DEFECTIVE 19*
WPM	McCown Woody Plant Medium
