# Supplementary material for: Evaluating the Efficiency of gRNAs in CRISPR/Cas9 Mediated Genome Editing in Poplars
Source: Int J Mol Sci. 2019 Jul 24;20(15):3623. doi: 10.3390/ijms20153623 (PMC6696231; doi:10.3390/ijms20153623)
Supplement: Supplementary file 1 [file ijms-20-03623-s001.zip › ijms-543174-supplementary/Online Resource_1 gRNA sequences and structures.pdf]

T. Bruegmann*, K. Deecke, M. Fladung*
Thuenen Institute of Forest Genetics, Grosshansdorf, Germany
tobias.bruegmann@thuenen.de; matthias.fladung@thuenen.de

# Online Resource 1
# gRNA sequences and structures

**The given sequences are colored in the following scheme: gRNA sequence green, hairpin sequence blue, tracrRNA grey.**

gRNA1 sequence (targeting *SOC1*)
UUGGACAGCC ACAGAUGCGC GUUUUAGAGC UAGAAAUAGC AAGUUAAAAU AAGGCUAGUC
CGUUAUCAAC UUGAAAAAGU GGCACCGAGU CGGUGCUUUU UUU

gRNA2 sequence (targeting *SOC1* Paralog 1 and Paralog 2)
GUAAAUGCAU CUUCCUUCAC GUUUUAGAGC UAGAAAUAGC AAGUUAAAAU AAGGCUAGUC
CGUUAUCAAC UUGAAAAAGU GGCACCGAGU CGGUGCUUUU UUU

gRNA3 sequence (targeting *AGL8.1*)
UUCCCGAAUG AGUUCAAGGG GUUUUAGAGC UAGAAAUAGC AAGUUAAAAU AAGGCUAGUC
CGUUAUCAAC UUGAAAAAGU GGCACCGAGU CGGUGCUUUU UUU

gRNA4 sequence (targeting *AGL8.2*)
UGCCCGAAUG UGUUUAAGGG GUUUUAGAGC UAGAAAUAGC AAGUUAAAAU AAGGCUAGUC
CGUUAUCAAC UUGAAAAAGU GGCACCGAGU CGGUGCUUUU UUU

gRNA5 sequence (targeting *NFP-like1*)
AGUUGAUUUG GAAAUAAUGG UUUUAGAGCU AGAAAUAGCA AGUUAAAAUA AGGCUAGUCC
GUUAUCAACU UGAAAAAGUG GCACCGAGUC GGUGCUUUUU UU

gRNA6 sequence (targeting *NFP-like1*)
UUCUUCUCGA UUCCACAUUU GUUUUAGAGC UAGAAAUAGC AAGUUAAAAU AAGGCUAGUC
CGUUAUCAAC UUGAAAAAGU GGCACCGAGU CGGUGCUUUU UUU

gRNA7 sequence (targeting *NFP-like3* and *NFP-like4*)
CGAGAAAAGG UCACCGAUUG GUUUUAGAGC UAGAAAUAGC AAGUUAAAAU AAGGCUAGUC
CGUUAUCAAC UUGAAAAAGU GGCACCGAGU CGGUGCUUUU UUU

gRNA8 sequence (targeting *NFP-like3* and *NFP-like4*)
UACUUGGUUU UGAUAUCAUA GUUUUAGAGC UAGAAAUAGC AAGUUAAAAU AAGGCUAGUC
CGUUAUCAAC UUGAAAAAGU GGCACCGAGU CGGUGCUUUU UUU

gRNA9 sequence (targeting *TOZ19*)
UCCAGAAGCA UGGCAAGCCA GUUUUAGAGC UAGAAAUAGC AAGUUAAAAU AAGGCUAGUC
CGUUAUCAAC UUGAAAAAGU GGCACCGAGU CGGUGCUUUU UUU

**Evaluating the Efficiency of gRNAs in CRISPR/Cas9 Mediated Genome Editing in Poplars**
T. Bruegmann*, K. Deecke, M. Fladung*
Thuenen Institute of Forest Genetics, Grosshansdorf, Germany
tobias.bruegmann@thuenen.de; matthias.fladung@thuenen.de

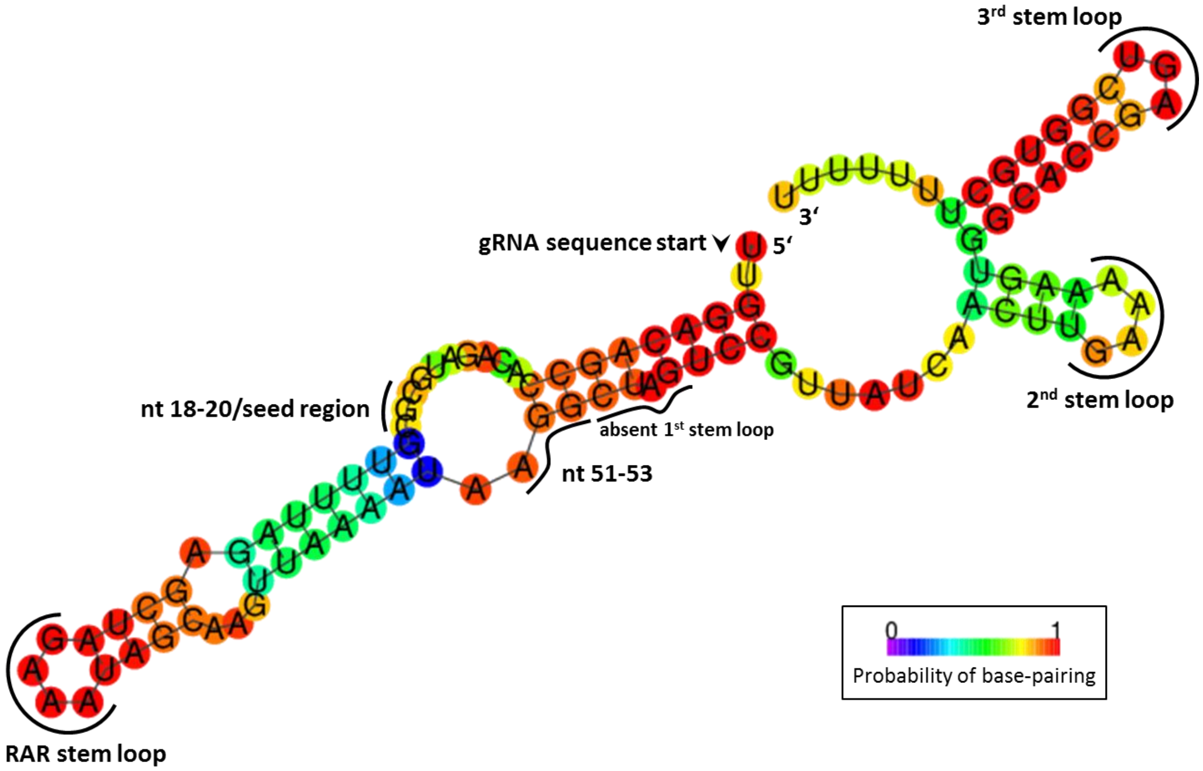


gRNA1

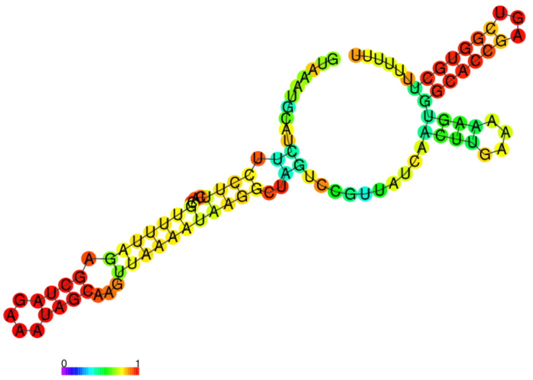

gRNA2

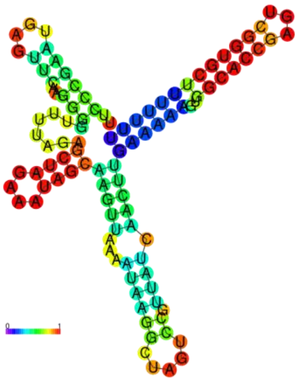

gRNA3

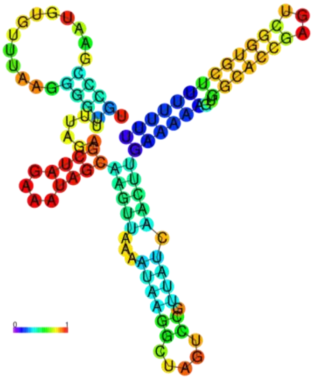

gRNA4

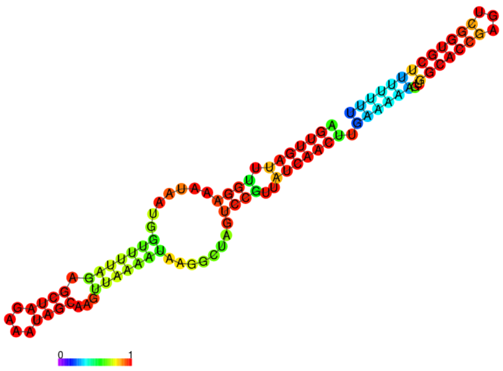

gRNA5

**Evaluating the Efficiency of gRNAs in CRISPR/Cas9 Mediated Genome Editing in Poplars**

T. Bruegmann*, K. Deecke, M. Fladung*
Thuenen Institute of Forest Genetics, Grosshansdorf, Germany
tobias.bruegmann@thuenen.de; matthias.fladung@thuenen.de

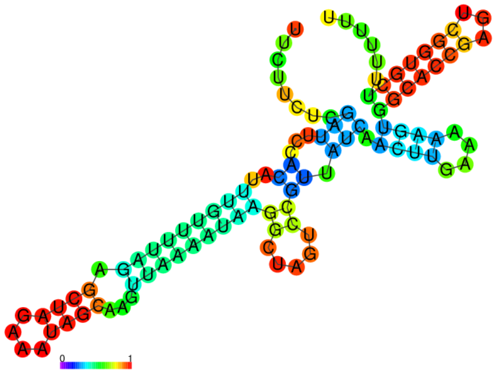

gRNA6

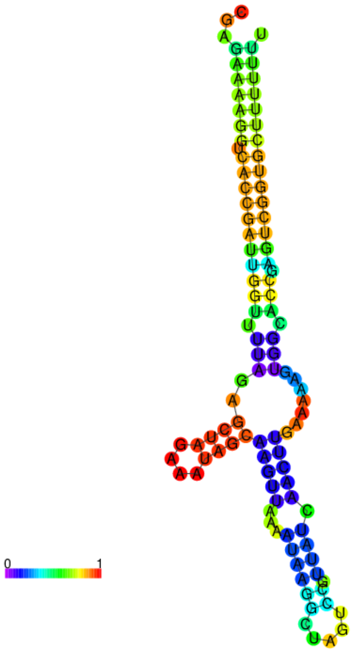

gRNA7

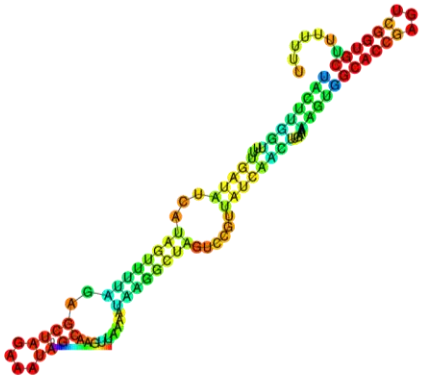

gRNA8

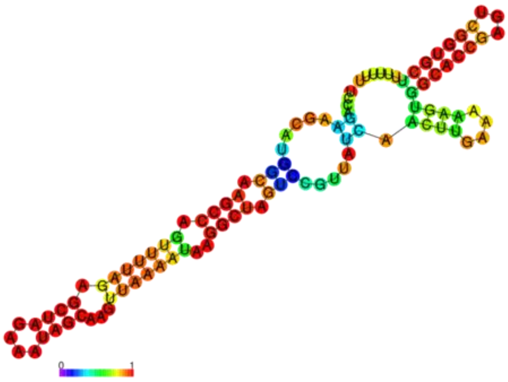

gRNA9
