# Supplementary material for: Evaluating the Efficiency of gRNAs in CRISPR/Cas9 Mediated Genome Editing in Poplars
Source: Int J Mol Sci. 2019 Jul 24;20(15):3623. doi: 10.3390/ijms20153623 (PMC6696231; doi:10.3390/ijms20153623)
Supplement: Supplementary file 1 [file ijms-20-03623-s001.zip › ijms-543174-supplementary/Online Resource_2 NFP-like1 gRNA5 at first time point.pdf]

Evaluating the Efficiency of gRNAs in CRISPR/Cas9 Mediated Genome Editing in Poplars
T. Bruegmann*, K. Deecke, M. Fladung*
Thuenen Institute of Forest Genetics, Grosshansdorf, Germany
tobias.bruegmann@thuenen.de; matthias.fladung@thuenen.de

Online Resource 2
Sequencing results of *NFP-like1* at first time point

Modification in *NFP-like1* gRNA5 at first time point. Highlighted in yellow: PAM-recognition motif. Letter in red represent insertions according to wildtype. Gaps in red highlighted represent deletions according to wildtype. Sequences illustrate both alleles.

Plant line	Sequencing results	Commentary
Wildtype	...GGAAATA-ATGGGG...<br>...GGAAATA-ATGGGG...	
N 481-2	...GGAAATA--TGGGG...<br>...GGAAATA--TGGGG...	Homozygous deletion of A
N 481-9	...GGAA------GGGG...<br>...GGAAATA--TGGGG...	Biallelic mutation: deletion ATAAT and deletion A
N 481-15	...GGAAATA--TGGGG...<br>...GGAAATAAATGGGG...	Biallelic mutation: deletion A, insertion A
N 481-23	...GGAAA---ATGGGG...<br>...GGAAATA--TGGGG...<br>...GGAAA----TGGGG...	Chimera: deletion TA, deletion A, deletion TAA
N 481-33	...GGAAATAAATGGGG...<br>...GGAAATA--TGGGG...	Biallelic mutation: insertion A, deletion A
N 481-97	...GGAATA(36nt)ATGGGG...<br>...GGAATA(42nt)ATGGGG...	Biallelic mutation: insertion 36 nt, insertion 42 nt
