# Supplementary material for: Evaluating the Efficiency of gRNAs in CRISPR/Cas9 Mediated Genome Editing in Poplars
Source: Int J Mol Sci. 2019 Jul 24;20(15):3623. doi: 10.3390/ijms20153623 (PMC6696231; doi:10.3390/ijms20153623)
Supplement: Supplementary file 1 [file ijms-20-03623-s001.zip › ijms-543174-supplementary/Online Resource_3 Transformation vectors.pdf]

Evaluating the Efficiency of gRNAs in CRISPR/Cas9 Mediated Genome Editing in Poplars
T. Bruegmann*, K. Deecke, M. Fladung*
Thuenen Institute of Forest Genetics, Grosshansdorf, Germany
tobias.bruegmann@thuenen.de; matthias.fladung@thuenen.de

Online Resource 3
Transformation vectors

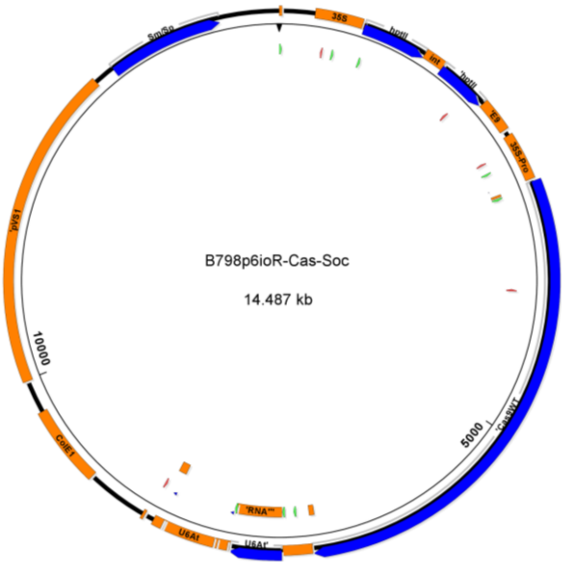

Fig. 1 B798p6ioR-Cas-Soc plasmid encoding gRNAs for the knockout of *SOC1* and two *SOC1* paralogous genes

35S: cauliflower mosaic virus 35S promoter; int: StLS1 (*Solanum tuberosum* rbcL-large subunit 1)-intron; hptII: coding sequence of hygromycin resistance gene; ‘E9: terminator Rbcs-E9; 35S-Pro: cauliflower mosaic virus 35S promoter region; ‘Cas9WT: Cas9 coding sequence of Cas9 wildtype gene (*Streptococcus pyogenes*); U6at: coding U6 promoter (*Arabidopsis thaliana*) followed by gRNA sequence; ColE1: origin of replication; ‘pVS1: origin of replication in *A. tumefaciens*; Sm/Sp: coding sequence of streptomycin/spectinomycin resistance gene

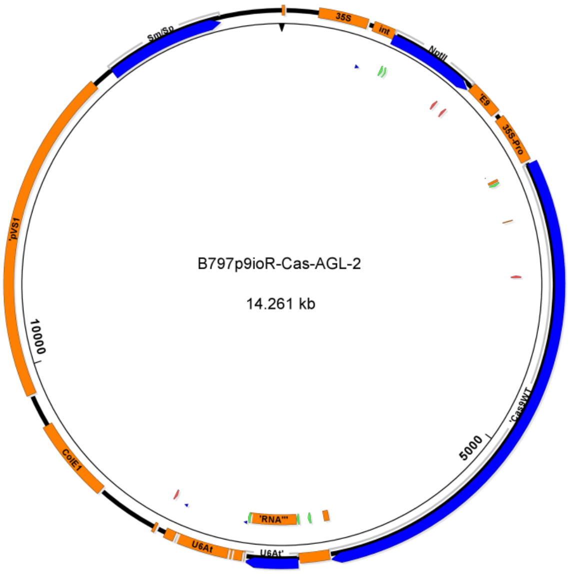

Fig. 2 B797p9ioR-Cas-AGL-2 plasmid encoding gRNAs for the knockout of *AGL8.1* and *AGL8.2*

35S: cauliflower mosaic virus 35S promoter; int: StLS1 (*Solanum tuberosum* rbcL-large subunit 1)-intron; NptII: coding sequence of kanamycin resistance gene; ‘E9: terminator Rbcs-E9; 35S-Pro: cauliflower mosaic virus 35S promoter region; ‘Cas9WT: Cas9 coding sequence of Cas9 wildtype gene (*Streptococcus pyogenes*); U6at: coding U6 promoter (*Arabidopsis thaliana*) followed by gRNA sequence; ColE1: origin of replication; ‘pVS1: origin of replication in *A. tumefaciens*; Sm/Sp: coding sequence of streptomycin/spectinomycin resistance gene

Evaluating the Efficiency of gRNAs in CRISPR/Cas9 Mediated Genome Editing in Poplars
T. Bruegmann*, K. Deecke, M. Fladung*
Thuenen Institute of Forest Genetics, Grosshansdorf, Germany
tobias.bruegmann@thuenen.de; matthias.fladung@thuenen.de

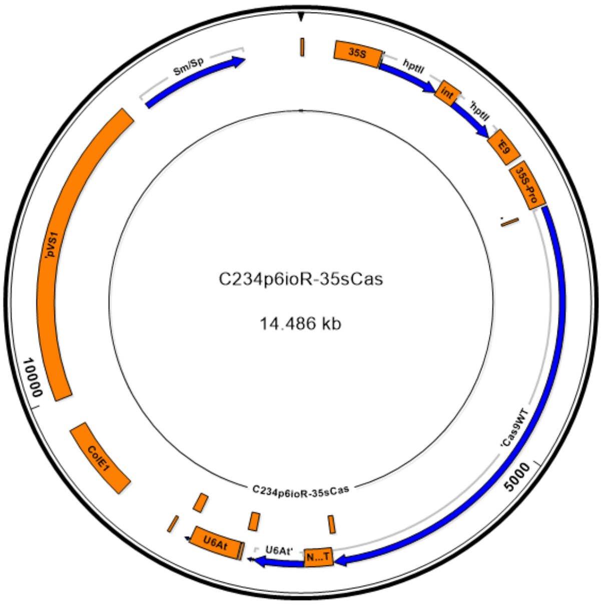

Fig. 3 C234p6ioR-35sCas plasmid for knockout of both *NFP-like1* and *NFP-like2* genes

35S: cauliflower mosaic virus 35S promoter; hptII: coding sequence of hygromycin resistance gene; Int: StLS1 (*Solanum tuberosum* rbcL-large subunit 1)-intron; 'E9: terminator Rbcs-E9; 35S-Pro: cauliflower mosaic virus 35S promoter region; 'Cas9WT: Cas9 coding sequence of Cas9 wildtype gene (*Streptococcus pyogenes*); N…T: nopaline synthase terminator; U6At': coding U6 promoter (*Arabidopsis thaliana*) followed by gRNA sequence; U6At: U6 terminator (*Arabidopsis thaliana*); ColE1: origin of replication; 'pVS1: origin of replication in *A. tumefaciens*; Sm/Sp: coding sequence of streptomycin/spectinomycin resistance gene

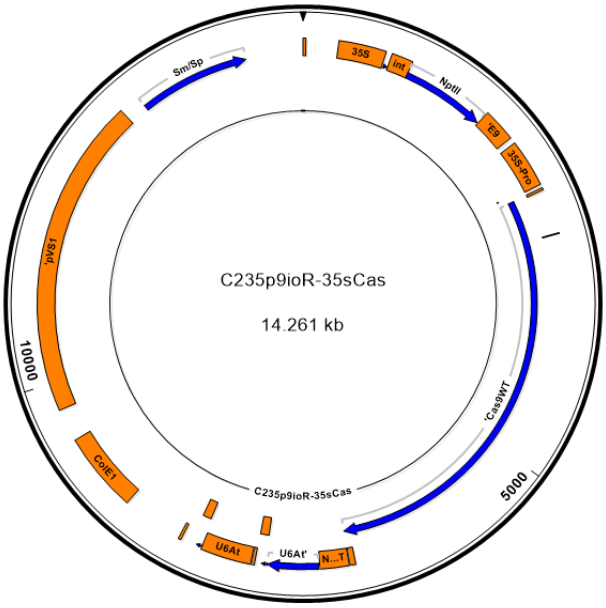

Fig. 4 C235p9ioR-35sCas plasmid for knockout of both *NFP-like3* and *NFP-like4* genes.

35S: cauliflower mosaic virus 35S promoter; Int: StLS1 (*Solanum tuberosum* rbcL-large subunit 1)-intron; NptII: coding sequence of kanamycin resistance gene; 'E9: terminator Rbcs-E9; 35S-Pro: cauliflower mosaic virus 35S promoter region; 'Cas9WT: Cas9 coding sequence of Cas9 wildtype gene (*Streptococcus pyogenes*); N…T: nopaline synthase terminator; U6at: coding U6 promoter (*Arabidopsis thaliana*) followed by gRNA sequence; ColE1: origin of replication; 'pVS1: origin of replication in *A. tumefaciens*; Sm/Sp: coding sequence of streptomycin/spectinomycin resistance gene

Evaluating the Efficiency of gRNAs in CRISPR/Cas9 Mediated Genome Editing in Poplars
T. Bruegmann*, K. Deecke, M. Fladung*
Thuenen Institute of Forest Genetics, Grosshansdorf, Germany
tobias.bruegmann@thuenen.de; matthias.fladung@thuenen.de

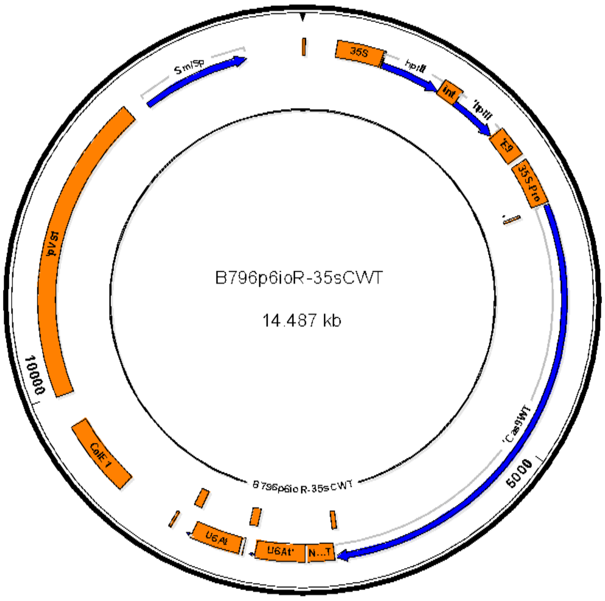

Fig. 5 B796p6ioR-35sCWT plasmid for knockout of the *TOZ19* gene

LB: left border of T-DNA; 35S: cauliflower mosaic virus 35S promoter; Int: StLS1 (*Solanum tuberosum* rbcL-large subunit 1)-intron; hptII: coding sequence of the hygromycin resistence gene; `E9: terminator; 35S-Pro: cauliflower mosaic virus 35S promotor; `Cas9WT: coding sequence of Cas9-WT gene; U6At/U6At`: sequence of gRNA; RB: right border of T-DNA; ColE1: origin of replication; `pVS1: origin of replication in *A. tumefaciens*; Sm/Sp: coding sequence of streptomycin/spectinomycin resistance gene.

Cas9 sequence including NLS

CCCAAGAAGA AGCGCAAAGT GGGAATCCAC GGAGCTCCAG ACAAGAAGTA CAGCATTGGT CTGGACATCG GGACCAACTC CGTCGGCTGG
GCCGTTATCA CCGATGAGTA CAAAGTGCCT TCTAAGAAGT TCAAAGTCCT CGGAAACACT GACAGGCACA GCATCAAGAA GAACTTGATT
GGCGCCCTTC TCTTCGACTC CGGCGAAACC GCTGAGGCCA CCCGTCTGAA GCGCACCGCC AGAAGGCGCT ACACCCGTCG CAAGAATAGG
ATCTGCTACC TCCAGGAGAT CTTCAGCAAC GAGATGGCCA AAGTGGACGA TTCCTTCTTT CACCGTTTGG AGGAGAGCTT CCTTGTCGAG
GAGGACAAGA AGCACGAGCG CCATCCCATC TTCGGAAACA TTGTTGACGA GGTGGCTTAC CACGAGAAGT ATCCAACTAT CTACCACCTC
AGAAAGAAGC TGGTGGACTC CACCGACAAG GCCGATTTGA GGCTCATCTA CCTTGCCCTC GCTCACATGA TCAAGTTCCG CGGTCACTTC
CTGATTGAGG GCGACCTCAA CCCCGACAAC TCTGACGTCG ATAAGTTGTT CATCCAACTG GTCCAGACCT ACAACCAGCT TTTCGAGGAG
AATCCTATCA ACGCCTCCGG GGTTGACGCC AAGGCTATTC TCAGCGCCCG TCTGTCCAAG AGCCGCAGGC TTGAGAACTT GATCGCCCAG
CTCCCAGGAG AGAAGAAGAA CGGCCTCTTC GGAAACCTGA TCGCTCTCTC CTTGGGCCTT ACCCCTAACT TCAAGTCTAA TTTTGACCTC
GCCGAGGACG CCAAGCTGCA ACTCAGCAAG GACACCTACG ATGACGACTT GGACAACCTG CTCGCCCAGA TCGGCGATCA GTACGCTGAC
CTTTTCTTGG CCGCCAAGAA CCTCTCCGAC GCTATTCTGC TCTCCGACAT CCTTCGCGTG AACACTGAGA TCACCAAGGC CCCACTGAGC
GCCTCCATGA TCAAGCGTTA CGACGAACAC CACCAAGATT TGACCCTCCT CAAGGCTCTT GTCAGACAGC AGCTGCCCGA GAAGTACAAG
GAGATTTTCT TCGACCAGAG CAAGAACGGA TACGCCGGTT ACATCGACGG CGGGGCCTCC CAAGAGGAGT TCTACAAGTT CATCAAGCCC
ATCTTGGAGA AGATGGACGG CACCGAAGAG CTGCTCGTGA AGCTCAACCG CGAGGATCTT TTGAGGAAGC AGCGTACCTT CGACAACGGA
TCTATTCCCC ACCAGATCCA TCTCGGCGAG CTGCACGCTA TCCTCCGCAG GCAAGAGGAC TTCTATCCAT TCCTTAAGGA CAATCGCGAG
AAGATTGAAA AGATCCTCAC TTTTAGAATC CCTTACTACG TCGGACCCTT GGCCCGTGGC AACAGCCGCT TCGCCTGGAT GACCAGGAAG
TCCGAGGAAA CCATCACCCC ATGGAACTTC GAGGAGGTGG TTGACAAGGG GGCCAGCGCT CAGTCCTTCA TTGAGCGCAT GACCAACTTC
GATAAGAACC TGCCCAACGA GAAAGTCCTC CCAAAGCACT CCCTTCTGTA CGAGTACTTC ACTGTCTACA ATGAGTTGAC CAAAGTGAAG
TACGTGACCG AGGGTATGCG TAAGCCTGCC TTCCTCAGCG GCGAGCAGAA GAAGGCCATC GTTGACCTCC TGTTCAAGAC CAACAGGAAA
GTCACCGTGA AGCAGCTTAA GGAGGACTAC TTCAAGAAGA TCGAGTGCTT TGACTCTGTC GAGATCAGCG GAGTGGAGGA TCGCTTCAAC
GCTTCCTTGG GCACTTACCA CGACCTCCTG AAGATTATCA AGGACAAGGA CTTCCTCGAC AACGAGGAGA ACGAAGATAT CTTGGAGGAC
ATCGTTCTTA CCCTCACCCT GTTCGAGGAC AGAGAGATGA TTGAGGAACG CCTCAAGACC TACGCCCACT TGTTCGACGA TAAAGTCATG
AAGCAACTTA AGCGTAGGCG CTACACCGGA TGGGGCAGGC TGTCCCGTAA GCTCATCAAC GGTATCCGCG ACAAGCAGAG CGGCAAGACT
ATTCTCGACT TCTTGAAGTC CGACGGATTC GCCAATCGCA ACTTCATGCA GCTGATCCAC GACGATTCTC TTACCTTCAA GGAGGACATC
CAAAAGGCTC AAGTGTCCGG CCAGGGCGAC AGCCTCCACG AGCACATCGC CAACCTGGCC GGGAGCCCCG CTATTAAGAA GGGAATCCTC
CAGACCGTCA AGGTGGTTGA CGAGTTGGTC AAGGTGATGG GCAGACATAA GCCAGAGAAC ATCGTCATCG AGATGGCCAG GGAAAACCAG
ACCACCCAGA AGGGACAGAA GAACTCCCGT GAGCGCATGA AGAGGATTGA GGAGGGCATC AAGGAGCTTG GTTCCCAGAT CCTCAAGGAG
CACCCCGTGG AAAACACCCA ACTCCAGAAT GAGAAGCTGT ATTTGTACTA CCTTCAGAAC GGGCGCGATA TGTACGTTGA CCAAGAGCTG
GACATCAACC GCCTCAGCGA CTACGACGTG GATCACATTG TCCCTCAGTC CTTTCTCAAG GACGACTCTA TCGACAACAA GGTGTTGACT
AGAAGCGATA AGAACCGCGG CAAGTCCGAC AACGTCCCAT CCGAGGAGGT GGTCAAGAAG ATGAAGAATT ACTGGAGGCA GCTGCTTAAC

Evaluating the Efficiency of gRNAs in CRISPR/Cas9 Mediated Genome Editing in Poplars

T. Bruegmann*, K. Deecke, M. Fladung*
Thuenen Institute of Forest Genetics, Grosshansdorf, Germany
tobias.bruegmann@thuenen.de; matthias.fladung@thuenen.de

```
GCCAAGCTCA TCACCCAGCG CAAGTTCGAC AACTTGACCA AGGCCGAGCG TGGAGGCCTC AGCGAACTGG ACAAGGCTGG ATTCATCAAG
AGGCAACTTG TTGAAACCCG CCAGATTACC AAGCACGTGG CCCAGATCCT CGACTCCCGT ATGAACACTA AGTACGATGA GAACGACAAG
CTGATCCGCG AGGTCAAAGT GATTACCCTC AAGAGCAAGC TCGTGTCTGA CTTCAGAAAG GACTTCCAAT TCTACAAGGT TAGGGAGATC
AACAACTACC ACCACGCCCA CGATGCTTAC CTTAATGCCG TGGTCGGCAC CGCCTTGATC AAGAAGTACC CCAAGCTGGA GTCCGAGTTC
GTGTATGGTG ACTACAAGGT CTACGACGTT CGCAAGATGA TCGCTAAGAG CGAGCAGGAG ATTGGCAAGG CCACCGCCAA GTACTTCTTC
TACTCCAACA TCATGAACTT CTTTAAGACC GAGATCACTT TGGCTAACGG GGAGATCCGT AAGCGCCCTC TCATTGAAAC CAACGGAGAA
ACCGGCGAGA TCGTGTGGGA CAAGGGCAGG GACTTCGCCA CCGTCAGAAA AGTGCTGAGC ATGCCCCAAG TCAACATCGT GAAGAAAACC
GAGGTTCAGA CTGGAGGCTT CTCCAAGGAG TCTATCCTCC CAAAGCGCAA TTCCGATAAG TTGATTGCCC GTAAGAAGGA CTGGGACCCC
AAGAAGTACG GTGGATTCGA CAGCCCAACC GTCGCCTACT CCGTGCTTGT CGTGGCTAAA GTTGAGAAGG GCAAGAGCAA GAAGCTCAAG
TCCGTCAAGG AGCTGCTCGG GATCACCATC ATGGAGCGCA GCTCCTTCGA GAAGAACCCT ATTGATTTCC TTGAGGCCAA GGGCTACAAG
GAAGTGAAGA AGGACTTGAT CATCAAGCTC CCCAAGTACT CTCTGTTCGA GCTTGAGAAC GGAAGGAAGC GTATGCTCGC CTCCGCTGGC
GAGCTGCAAA AGGGAAACGA GTTGGCCCTC CCAAGCAAGT ACGTCAACTT CCTGTACCTC GCCTCCCACT ATGAGAAGCT CAAGGGCAGC
CCCGAGGACA ACGAACAGAA GCAGTTGTTT GTGGAGCAGC ATAAGCACTA CCTTGACGAG ATCATTGAGC AGATCAGCGA GTTCTCCAAG
CGCGTTATCC TGGCTGACGC CAATCTCGAT AAAGTCCTTT CTGCCTACAA CAAGCACAGG GACAAGCCTA TCAGAGAGCA GGCTGAAAAC
ATTATCCACT TGTTCACCCT CACCAACCTG GGTGCCCCAG CCGCCTTCAA GTACTTCGAC ACTACCATCG ACCGCAAGCG TTACACCTCC
ACCAAGGAAG TGCTCGATGC TACCCTTATC CACCAGAGCA TTACTGGGTT GTACGAAACC AGGATCGACC TGTCCCAACT CGGCGGAGAC
AAGCGCCCCG CCGCCACCAA GAAGGCTGGC CAGGCCAAGA AGAAGAAGTA A
```

NLS sequence in green
