# Supplementary material for: Evaluating the Efficiency of gRNAs in CRISPR/Cas9 Mediated Genome Editing in Poplars
Source: Int J Mol Sci. 2019 Jul 24;20(15):3623. doi: 10.3390/ijms20153623 (PMC6696231; doi:10.3390/ijms20153623)
Supplement: Supplementary file 1 [file ijms-20-03623-s001.zip › ijms-543174-supplementary/Online Resource_4 PCR and sequencing primers.pdf]

Evaluating the Efficiency of gRNAs in CRISPR/Cas9 Mediated Genome Editing in Poplars
T. Bruegmann*, K. Deecke, M. Fladung*
Thuenen Institute of Forest Genetics, Grosshansdorf, Germany
tobias.bruegmann@thuenen.de; matthias.fladung@thuenen.de

Online Resource 4
PCR and sequencing primers

PCR and sequencing primers for proof of mutation in gRNA targeting genomic sites of the candidate genes used in this study. T_A: annealing temperature; F: forward sequence; R: reverse sequence

Target	Amplification Primer	T_A	Sequencing Primer
1	F: 5‘-AGA CTC AGT TAT TCA AGG AGC-3‘<br>R: 5‘-TAT GAG GAC ACT ATA TCG TTG-3‘	53 °C	R: 5‘-TAT GAG GAC ACT ATA TCG TTG-3‘
2	F: 5‘-GTG AAT CCA GTG TAT GAT CCT G-3‘<br>R: 5‘-TGG TTA TGG GAT AGG CGT G-3‘	55 °C	F: 5‘-GGC CAT ACC AAA GGC TTC AAT G-3‘
3	F: 5‘-GGC CAT ACC AAA GTC TTC ACT G-3‘<br>R: 5‘-CTC TCC TAA GAG CTT CCT GC-3‘	57 °C	F: 5‘-GGC CAT ACC AAA GTC TTC ACT G-3‘
4	F: 5‘-GAA GCA TCG TCT TGT TGT GC-3‘<br>R: 5‘-CTG CAG CTC AGA AAT CGA CTG-3‘	57 °C	F: 5‘-CTC CGA ACT TGA TTG CCT GC-3‘<br>R: 5‘-CTG CAG CTC AGA AAT CGA CTG-3‘
5	F: 5‘-TAG AGA ATT CGT ACA TCA GCT-3‘<br>R: 5‘-CTG TAG GAG TAT TTA GTA TGC-3‘	52 °C	F: 5‘-GGT ACT GAC TTG ATT GCC TGC-3‘
6	F: 5‘-TTT CCA CAC AGG CTC TTC TTC TC-3‘<br>R: 5‘-CTG TTT CCA TCC CAG ATG TCC-3‘<br><br><br><br><br>F: 5’-GTC CAC CCA TGTC TCC TTA-3’<br>R: 5’- TAG TGG TTT TCA ATC ACA ATG T-3’	60 °C<br><br><br><br><br><br>58 °C	For the editing region of gRNA5:<br>F, T1: 5‘-TTT CCA CAC AGG CTC TTC TTC TC-3‘<br>F, T2: 5‘-AAA ATC AAT GGC CGG GTC TTG G-3‘<br>For the editing region of gRNA6:<br>R, T1: 5‘-GTA TTT GAA GGG CAC TTA CAG AAC-3‘<br>R, T2: 5‘-CTG TTT CCA TCC CAG ATG TCC-3‘<br>For the editing region of gRNA5<br>F: 5’-GTC CAC CCA TGT CTC CTT A-3’<br>R: 5’- TAG TGG TTT TCA ATC ACA ATG T-3’
7	F: 5‘-ACA AAA CAT TGC AAG GCA GAA AAG-3‘<br>R: 5‘-CCC ACT TAC CAA GAC CAG TAA AA-3‘	61.1 °C	F: 5‘-ACA GTA ATA TCC TCC ACC ATT-3‘<br>R: 5‘-AGT GTT GCC TGC CTC GAT GGT-3‘
8	F: 5‘-AGA AAG GCA GAA CAA CCA AG-3‘<br>R: 5‘-TAT GTT ATT GCC ATT AGC ATC CAC-3‘	52.2 °C	F: 5‘-CTA TTA CAG TAC TAT CCT CCA-3‘<br>R: 5‘-AAG TAT TCA GTT GAG ACA AGG-3‘
9	F: 5’-ACT TGT CCA CTT TCA AAT GC -3’<br>R: 5’-CAC GTT CAC CAA CTG TGA TAA AAT-3’	58 °C	F. 5’-ACT TGT CCA CTT TCA AAT GC-3’<br>R: 5’-CAC GTT CAC CAA CTG TGA TAA AAT-3’
